# Are Resistance Training-Induced BDNF in Hemodialysis Patients Associated with Depressive Symptoms, Quality of Life, Antioxidant Capacity, and Muscle Strength? An Insight for the Muscle–Brain–Renal Axis

**DOI:** 10.3390/ijerph182111299

**Published:** 2021-10-27

**Authors:** Lysleine Alves Deus, Hugo de Luca Corrêa, Rodrigo Vanerson Passos Neves, Andrea Lucena Reis, Fernando Sousa Honorato, Victor Lopes Silva, Michel Kendy Souza, Thaís Branquinho de Araújo, Lucas Santos de Gusmão Alves, Caio Victor Sousa, Thaís Lucena Reis, Lucas Soares de Aguiar, Herbert Gustavo Simões, Jonato Prestes, Gislane Ferreira Melo, Thiago Santos Rosa

**Affiliations:** 1 Graduate Program in Physical Education, Catholic University of Brasília, Brasília 71966-700, Brazil; hugo.efucb@gmail.com (H.d.L.C.); rpassosneves@yahoo.com.br (R.V.P.N.); andrealucereis@gmail.com (A.L.R.); ferhon@gmail.com (F.S.H.); victornutri@yahoo.com.br (V.L.S.); thaisbranquinhodearaujo@gmail.com (T.B.d.A.); hgsimoes@gmail.com (H.G.S.); jonatop@gmail.com (J.P.); gislane.melo@gmail.com (G.F.M.); thiagoacsdkp@gmail.com (T.S.R.); 2 Department of Nephrology, Federal University of São Paulo, São Paulo 04021-001, Brazil; mks_gtr@hotmail.com; 3 Graduate Program in Medicine, Catholic University of Brasília, Brasília 71966-700, Brazil; lucasgusmao1204@gmail.com; 4 Bouve College of Health Sciences, Northeastern University, Boston, MA 02115, USA; c.sousa@northeastern.edu; 5 Medical Graduate Program, University of Brasília, Brasília 70910-900, Brazil; thaisbrasilia@gmail.com (T.L.R.); lucas.saguiar.97@gmail.com (L.S.d.A.)

**Keywords:** quality of life, SF-36, redox balance, neuroprotection, depression

## Abstract

Background: Hemodialysis patients are suffering from depressive symptoms. Brain-derived neurotrophic factor (BDNF) levels are negatively associated with depressive symptoms and decrease during a single hemodialysis session. Resistance training (RT) might be an additional non-pharmacological tool to increase BDNF and promote mental health. Methods: Two randomized groups of hemodialysis patients: control (CTL, *n* = 76/F36; 66.33 ± 3.88 years) and RT (*n* = 81/F35; 67.27 ± 3.24 years). RT completed six months of training thrice a week under the supervision of strength and conditioning professional immediately before the dialysis session. Training loads were adjusted using the OMNI rating of perceived exertion. The total antioxidant capacity (TROLOX), glutathione (GSH), thiobarbituric acid reactive substance (TBARS), and BDNF levels were analyzed in serum samples. Quality of life (assessed through Medical Outcomes—SF36), and Beck Depression Inventory was applied. Results: RT improved handgrip strength (21.17 ± 4.38 vs. 27.17 ± 4.34; *p =* 0.001) but not for CTL (20.09 ± 5.19 vs. 19.75 ± 5.54; *p =* 0.001). Post-training, RT group had higher values as compared to CTL related to TROLOX (RT,680.8 ± 225.2 vs. CTL,589.5 ± 195.9; *p =* 0.001) and GSH (RT, 9.33 ± 2.09 vs. CTL,5.00 ± 2.96; *p =* 0.001). RT group had lower values of TBARS as compared to CTL at post-training (RT, 11.06 ± 2.95 vs. CTL, 13.66 ± 2.62; *p =* 0.001). BDNF increased for RT (11.66 ± 5.20 vs. 19.60 ± 7.23; *p =* 0.001), but decreased for CTL (14.40 ± 4.99 vs. 10.84 ± 5.94; *p =* 0.001). Quality of life and mental health increased (*p =* 0.001) for RT, but did not change for CTL (*p =* 0.001). BDNF levels were associated with emotional dimensions of SF36, depressive symptoms, and handgrip (*p* = 0.001). Conclusions: RT was effective as a non-pharmacological tool to increased BDNF levels, quality of life, temper the redox balance and decrease depressive symptoms intensity in hemodialysis patients.

## 1. Introduction

Chronic kidney disease (CKD) is strongly associated with a low quality of life, increasing the prevalence of mental illness in this population [1,2]. Depressive symptoms rise according to CKD progression, underpinning a bridge between depression and a worse prognosis of hemodialysis patients [3]. Thus, strategies aiming to counteract or attenuate these factors are requested to avoid fatal outcomes, such as cessation of CKD treatment or suicide [4].

Low serum brain-derived neurotrophic factor (BDNF) levels are associated with depressive symptoms demonstrating its crucial role in mental health [5]. BDNF exerts antioxidant effects in neurons [2] protecting them from injury and disease [6]. According to previous studies, hemodialysis could decrease the serum concentration of BDNF [7]. In patients under dialysis, low serum BDNF level was associated with depressive symptoms and oxidative stress [8].

Exercising training is considered an effective non-pharmacological treatment that improves mental health and increases BDNF levels [9]. Moreover, it also tempers redox balance and inflammatory profile [10,11]. In this field, a growing body of evidence shows the effectiveness of resistance training (RT) as an additional treatment tool for patients with CKD at any stage. Recently, research showed that long-term RT performed by patients with CKD improved cardiac autonomic function, muscle strength, redox profile, and down-regulate inflammation-related markers [12,13,14]. However, whether RT can modulate BDNF in hemodialysis patients is unknown.

There is evidence showing that BDNF is correlated with higher handgrip strength [15]. Indeed, researchers may point out the interplay between muscle and brain, describing the BDNF-mediated redox regulation through the BDNF released from muscle contraction reaching the brain [16]. Moreover, evidence shows that BDNF is associated with sestrins [17], a biological mediator of exercise benefits, which its release can be increased through RT in patients under hemodialysis treatment [18,19].

Nevertheless, the interplay between resistance training, BDNF, depressive symptoms, and quality of life in patients with CKD is unknown. Taking the above mentioned into account, we hypothesized that RT would be effective to increase serum BDNF levels secondary to the improvement in the redox profile, quality of life, and mental health in patients with CKD. Therefore, we aimed to investigate the effect of long-term RT on: (i) BDNF levels, (ii) redox balance, (iii) quality of life, and (iv) depressive symptoms in patients with CKD under hemodialytic treatment. Confirmation of our hypothesis may allow future inferences about the muscle–brain–renal axis.

## 2. Methods

The university review board approved all experimental protocols under the number: 23007319.0.0000.0029. This study is part of a large trial registered on the Brazilian clinical trials registration: Available online: http://www.ensaiosclinicos.gov.br/rg/RBR-3gpg5w/, accessed on 28 July 2021, no. RBR-3gpg5w and also registered in the World Health Organization international clinical trial registry platform: Available online: http://apps.who.int/trialsearch/utn.aspx, no U1111-1237-8231 (30 July 2019) [12,13,14].

Throughout the study period, all patients were informed about the potential risks and benefits of their participation in this research study and freely signed the written informed consent form. They were accompanied and received the same recommendations from a multidisciplinary team composed of doctors, nurses, physiotherapists, and strength and conditioning professionals. Patients underwent all assessment procedures described below at baseline and post-training period.

### 2.1. Study Population

Patients undergoing maintenance hemodialysis were interviewed to participate in this clinical study. Two hundred two patients meet the eligibility criteria. Eligible participants were performing hemodialysis treatment for at least three months and three times per week, without complications from decompensated metabolic diseases (i.e., diabetic coma, ketoacidosis, hyperosmolar, and/or diabetes decompensated) evaluated by a nephrologist, in the last three months, except for vascular access correction. Exclusion criteria were as follows: recent acute myocardial infarction (within the past three months) or unstable angina; systemic lupus erythematosus; congenital kidney malformation or some autoimmune disease that affects the kidneys; osteoarticular complications that could compromise physical exercise; decompensated heart failure that could limit participation in training; severe decompensated diabetes; or severe neuropathy, retinopathy.

### 2.2. Randomization

**Figure 1:**
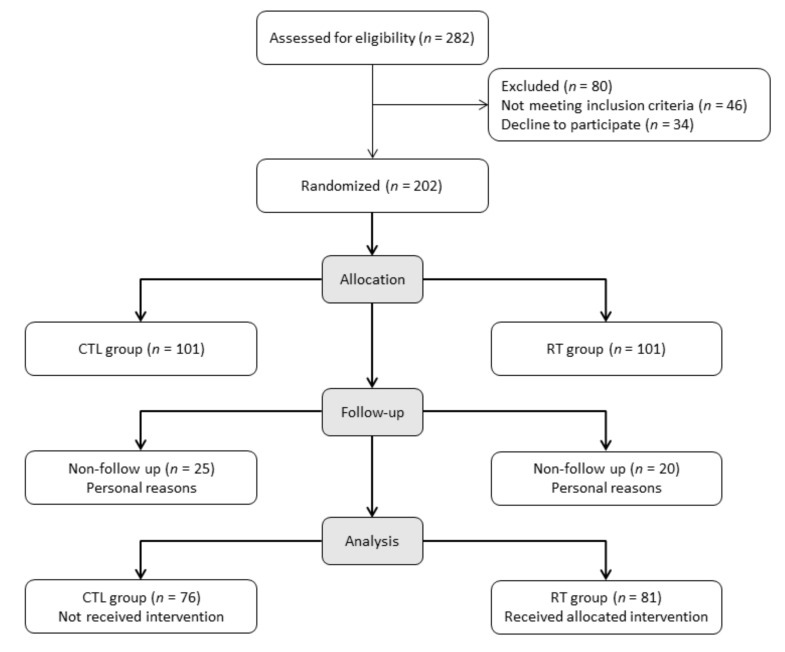
Participants flow-chart.

Initially, 202 male and female patients were randomized into two groups through a simple random number generation and allocated to the control group (CTL) and resistance training group (RT). Forty-five patients decline to participate due to personal reasons, among them twenty-five from the CTL group and twenty from the RT group. Finally, the total sample size was 157 patients (CTL, *n* = 76 and RT, *n* = 81) who participated in the present study. Women enrolled in this study were at the postmenopausal stage without any hormonal therapy. The numbers of women per group were CTL group (*n* = 36) and RT group (*n* = 35). Figure 1 presents the participants flow-chart.

### 2.3. Physical Assessment

Physical assessments consisted of weighed patients on a mechanical scale (Filizola^®^, São Paulo, Brazil) and measured their height with a stadiometer built into the scale. Then, body mass index was calculated by dividing body weight by the height squared (kg/m^2^).

### 2.4. Handgrip Strength

Handgrip strength was measured with a hydraulic hand dynamometer (Jamar^®^—Sammons Preston, Bolingbrook, IL, USA), following the American Society of Hand Therapists’ recommendations [20]. Measurements were performed with participants in sitting position, elbow joint at 90°, forearm in a neutral position, and wrist between 0° and 30° of extension. The average of three trials in the contralateral arm of the arteriovenous fistula was registered.

### 2.5. Blood Samples and Biochemical Analysis

Blood samples were drawn in the morning (between 7:00–8:00 am) after 8-h of fasting using a butterfly needle inserted into the antecubital vein and deposited in a 10 mL tube containing EDTA. The samples were centrifuged at 1500× *g* for 15 min, and the specimens were aliquoted into cryovials and stored at −80 °C.

BDNF (ng/mL): Serum BDNF levels were measured in triplicate using an enzyme-linked immunosorbent assay (ELISA) kit (Promega, Madison, WI, USA); inter- and intra-assay coefficients of variation were between 3% and 4.9%. Total antioxidant capacity (TROLOX; uM) was measured with a trolox-equivalent assay kit (QuantiChrom BioAssay Systems, California, CA, USA), using commercial kits and following the manufacturers’ protocols. Glutathione (GSH; µM) serum levels were measured according to the manufacturer’s instructions using the Glutathione Assay Kit (Sigma-Aldrich R., California, CA, USA). Thiobarbituric acid reactive substance (TBARS; nmol/mL) is one the most used methods to determine lipid peroxidation and oxidative damage in cells and tissues. The protocol used in the present study was adapted from Okawa [21]. Serum samples were diluted in 320 μL ultrapure H_2_O (1:5), and then 1 mL of trichloroacetic acid (TCA) 17.5%, pH 2.0 and 1 mL of thiobarbituric acid (TBA) 0.6%, pH 2.0 were added, respectively. After homogenization, the samples were kept in a water bath for 30 min at 95 °C. The reaction was interrupted with the immersion of the microtubes in ice, and the addition of 1 mL of TCA 70%, pH 2.0, and incubation for 20 min at room temperature. After centrifugation (3000 rpm for 15 min), the supernatant was moved to new microtubes for the spectrophotometry assessment at 540 nm. The concentration of lipid peroxidation products was calculated using the molar extinction coefficient equivalent for malondialdehyde (MDA-equivalent = 1.56 × 105 M^−1^ cm^−1^).

### 2.6. Quality of Life

Quality of life was assessed using the “Medical Outcomes Study 36 (SF36)” questionnaire [22]. The Medical Outcomes Study 36 (SF36) has been translated and validated into Portuguese by Ciconelli et al. [23] for the Brazilian population. A multidimensional questionnaire comprises 13 items, divided into eight dimensions: physical functioning, physical role, pain, general health, vitality, social function, emotional role, and emotional well-being. The results of each scale vary from 0 to 100 (worse to best possible status).

### 2.7. Beck Depression Inventory

Depression scores were assessed through the Beck Depression Inventory (BDI), a self-rating questionnaire for evaluating the severity of depression. It was created by [24], translated and validated into Portuguese by [25]. The questionnaire comprises 21 assertion groups, ranging from 0 to 3 related to the severity of the symptom. Each group consists of four self-evaluative statements of increasing severity. A total score of 0–9 indicates no depression, 10–15 mild to moderate depression, 16–23 moderate to severe depression, and a score of 24 or higher indicates severe depression.

### 2.8. Resistance Training Protocol

Patients completed one week of RT familiarization before initiating the RT protocol. RT was performed for six months, three times per week on non-consecutive days, under the supervision of strength and conditioning professional, and immediately before each dialysis session.

Patients completed twelve exercises each session which takes ~60 min (Table 1). They performed three sets of 8-to-12 repetitions resting for two minutes between sets and exercises. Repeats were set of the cadence of two seconds for concentric and eccentric muscle actions, respectively.

Training loads were monitored and adjusted using the OMNI-Resistance Exercise Scale (OMNI-RES) [26]. Initially, the training load corresponded to 8 repetitions at an RPE scale of 5 to 6 for the first 12 weeks, and 7 to 8 over the final 12 weeks (totaling 24 weeks of training). When the RPE indicated the load was too easy, the number of repetitions was increased. Then, if the participant exceeded 12 repetitions, the load was increased. Additionally, exercise techniques were maintained according to American College of Sports Medicine recommendations [27].

Furthermore, all patients were asked to maintain their habitual physical activity throughout the study, except for implementing the RT program.

Lastly, after the study, the exercise program was offered to all patients.

### 2.9. Statistical Analysis

A sample size of 157 participants provided a statistical power of 99% (1- β = 0.99), considering an alpha of 5% (α = 0.05) with a moderate effect size (f = 0.40). The Shapiro–Wilk test verified the normality of data. All variables were non-parametric. Then, Kruskal–Wallis with Dunn’s multiple comparisons test was applied. The significant level was set as 5% (*p =* 0.05), and data are expressed as mean, ± standard deviation, interquartile ranges, median, minimum and maximum. We further applied a Mann–Whitey test to verify possible differences among deltas, calculated as following ∆ = post-training—baseline to minimize type II error in comparisons. Finally, associations between variables were performed using Spearman’s correlation with a pooled of the groups. All statistical procedures were carried out using Graph Pad Prism (v6.0).

## 3. Results

There were no differences between groups related to time in hemodialysis (months), age, and body mass index at baseline. See Table 2 for mean, standard deviation, interquartile ranges, median, minimum, maximum, and statistical values.

RT group significantly improve handgrip strength (kgf) after the intervention, whereas their peers decreased (*p* < 0.0001; Table 3).

BDNF (ng/mL) levels decreased from baseline to post-training for CTL group but increased for the RT group (*p =* 0.0001). Post-training, RT group had higher levels than the CTL (*p =* 0.0001; Table 3).

There were no differences in TROLOX (uM) values from baseline to post-training for both groups. However, post-training, the RT group had higher values than the CTL (*p =* 0.0001; Table 3).

There was no difference in GSH (µM) values from baseline to post-training for the CTL group (*p =* 0.0001). However, RT increased GSH (µM) from baseline to post-training (*p =* 0.0001) and had higher values as compared to the CTL at post-training (*p =* 0.0001; Table 3).

TBARS (nmol/mL) values did not change from baseline to post-training for the CTL group (*p =* 0.0001). However, the RT group decreased TBARS (nmol/mL) from baseline to post-training (*p =* 0.0001) and had lower values as compared to the CTL at post-training (*p =* 0.0001; Table 3).

Regarding the quality of life, there were no differences from baseline to post-training for the CTL group (*p =* 0.0001). In contrast, RT group increased all dimensions values from baseline to post-training (*p =* 0.0001) and had higher values as compared to the CTL at post-training (*p =* 0.0001; Table 4).

The depression scores did not differ from baseline to post-training for both groups. However, the change in depression score was higher in RT compared to the CTL group (*p =* 0.0001; Table 5).

**Figure 2:**
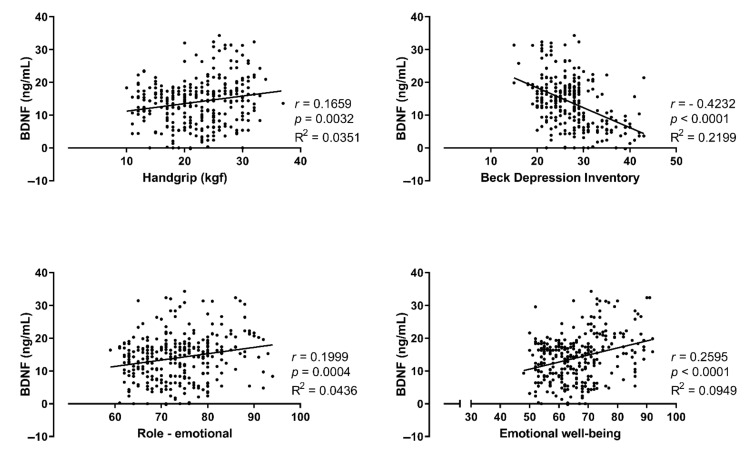
Spearman’s correlation with pooled groups (*n* = 157). Correlations between BDNF (ng/mL), handgrip (kgf), Beck depression inventory, and both dimension the emotional role and emotional well-being.

Additionally, Spearman’s correlation revealed a significant association between BDNF (ng/mL), handgrip (kgf), Beck depression inventory, and both dimensions of emotional role and emotional well-being. See Figure 2.

## 4. Discussion

This study is the first to assess the interplay between CKD, resistance training, and BDNF levels. Our main findings were: (i) RT improved handgrip strength; (ii) RT tempered the redox balance, increasing antioxidant defense (TROLOX and GSH) and decreasing pro-oxidative TBARS; (iii) RT improved serum BDNF levels; (iv) RT improved quality of life (assessed through Medical Outcomes Study 36—SF36); (v) Depressive symptoms intensity (assessed through Beck Depression Inventory—BDI); and (vi) serum BDNF levels were associated with role emotional, emotional well-being, Beck Depression Inventory, and handgrip strength. It is important to mention that data presented an associative, not causal, relationship between RT and serum BDNF levels.

The hemodialysis treatment induces sarcopenia, dynapenia [28], and negatively impacting the quality of life [29]. Noteworthy, low muscle strength is associated with elevated risk of all-cause mortality [30]. However, we observed that patients with CKD in hemodialysis treatment increased their muscle strength and improved quality of life parameters after RT.

Regarding the quality of life, it is noteworthy to emphasize that, during RT sessions, patients socialized with each other, shared experiences, and reported to have returned to perform the daily life activities that they were not able before training. This final aspect is likely another major aspect contributing to improving several components of the quality of life, which are physical function, physical role, pain, general health, vitality, social function, emotional role and emotional well-being. Perception strengthens by the correlations between emotional well-being, emotional rule, and depression symptoms scores with serum BDNF.

From a biochemical perspective, a persistent state of oxidative stress leads to low BDNF levels, increasing the vulnerability to depression symptoms, which could be treated by improving antioxidant defenses [31]. Evidence shows that RT has a great potential to enhance antioxidant defenses [13,32,33] and increase BDNF levels [16]. Currently, a study proposed the interplay between muscle and brain BDNF-mediated redox regulation. The authors describe that BDNF release from muscle contraction reaches the brain, activating multiple signaling pathways, such as Tropomyosin receptor kinase B (TrkB) and Nuclear factor-erythroid 2 related factor 2 (Nrf2), which regulates the expression of antioxidants molecules [16].

Though, the scientific discussion about the relationship between RT and BDNF is broad and reports contradictory outcomes. On one hand, RT did not affect BDNF levels [32,33]. On the other hand, research reports that RT induces an increase in BDNF levels [34]. This discrepancy may reflect the diversity in RT protocols related to duration, repetitions, sets, time of rest, the choice of exercises, weekly frequency [35]. In addition, some authors suggested that acute circulating BDNF regulation by exercise was dependent on the physical fitness level [11]. In this regard, a discerning view of previous studies reporting that RT did not affect BDNF levels reveals common characteristics: lower number of exercises (~7) and ≤12 weeks of intervention. These outcomes may give clues that a minimum RT volume threshold [36,37] and/or RT intensity at 12–14 (i.e., from somewhat hard to hard) RPE on the Borg Scale [38] may be required to improve BDNF levels. Reinforcing this hypothesis, in the present study, we showed that twelve exercises performed in the intensity of 5 to 6 for the first 12 weeks (i.e., somewhat hard), and 7–8 over the final 12 weeks (i.e., hard) RPE on the OMNI scale increased BDNF levels in hemodialysis patients. Finally, it is important to highlight that none of the mentioned studies assessed patients with CKD under hemodialysis treatment.

Moreover, knowing that a single hemodialysis session can decrease approximately 42% of the serum BDNF level [7], the time to perform the RT could be crucial. For instance, when the RT is applied before the hemodialysis session, it could give enough time to release and use the BDNF before its withdrawal by the hemodialysis process. Thus, previous reports that RT performed ~1 h before the hemodialysis session induced a significant increase in sestrins associated with BDNF levels [17,19].

Finally, individual responsiveness to RT should also be considered. Previous studies showed that older adults who participated in RT programs presented different responses in their BDNF levels, classifying them as lower or higher responders [39]. Furthermore, Forti et al. [40] reported that 12 weeks of RT increased BDNF levels in males but not in female participants. Otherwise, in the present study, there were no differences in serum BDNF levels between sexes. Therefore, although much has been written in this regard, caution must be taken to results interpretations.

The presented arguments are results from a relay on the available literature with speculative character. Even so, robust evidence demonstrated that RT has potent antioxidant, anti-inflammatory effects and a positive impact on the metabolism profile and muscle strength [12,14,28], which may contribute to BDNF levels enhancement, mental health, and quality of life of patients with CKD.

Despite planning and efforts to prevent limitations, we acknowledge there are opportunities for future investigations to improve. First, we identified an associative, not causal, relationship between RT and serum BDNF levels. We understood that the protocols which allow identified a causal relationship could be invasive and painful. The second limitation of the study is a lack of strict control of the patients’ eating habits. However, all patients received standard guidance concerning healthy eating habits.

Summarizing these findings, we observed that RT induces an increase in BDNF, antioxidant capacity, and quality of life, induced a change in depression symptoms in patients with CKD under hemodialysis treatment. Additionally, serum BDNF levels were associated with handgrip strength, role emotional and emotional well-being. Therefore, we suggest RT as a viable treatment to manage depressive symptoms, muscle strength, and quality of life in patients with CKD under hemodialysis treatment. Furthermore, we highlight BDNF as part of the mechanisms of exercise-induced benefits in this population, suggesting an important role of RT on the muscle–brain–renal axis.

## Figures and Tables

**Table 1:** Training characteristics.

Exercise	Instrument	Observations
1. Unilateral chest press	e-Lastic^®^	OMNI-RES 5 to 6 during 12 initial weeks. The exercise was performed with the contralateral arm to the fistula. A conservative measure intended to preserve arteriovenous fistula. The e-Lastic load record was the peak (maximum) load achieved in each movement to count the repetitions.
2. Squat	Bodyweight	OMNI-RES 7 to 8 during 12 last weeks From 4 to 15 reps—weekly increasing the number of reps At the beginning of the protocol, we encourage patients to perform few squats (4reps) but weekly increasing the number of reps.
3. Unilateral row	e-Lastic^®^	The exercise was performed with the contralateral arm to the fistula. A conservative measure intended to preserve arteriovenous fistula. The e-Lastic load record was the peak (maximum) load achieved in each movement to count the repetitions.
4. Bilateral knee extension	Weighted cuffs	Seated position Wrapped at the ankle.
5. Unilateral shoulder press	Dumbbells	Exercise performed with the arm contralateral to fistula. A conservative measure intended to preserve arteriovenous fistula.
6. Hip thrust	Weighted cuffs	Positioned at the hips.
7. Unilateral knee flexion	Weighted cuffs	Stand position Wrapped at the ankle.
8. Biceps curl	Dumbbells	The exercise was performed with the contralateral arm to the fistula. A conservative measure intended to preserve arteriovenous fistula.
9. Unilateral hip adduction	e-Lastic^®^	The e-Lastic load record was the peak (maximum) load achieved in each movement to count the repetitions.
10. Unilateral elbow extension	Dumbbells	The exercise was performed with the contralateral arm to the fistula. A conservative measure intended to preserve arteriovenous fistula.
11. Unilateral hip abduction	e-Lastic^®^	The e-Lastic load record was the peak (maximum) load achieved in each movement to count the repetitions.
12. Seated calf raise	Weighted cuffs	Wrapped across the quadriceps

**Table 2:** Baseline characteristics.

Variables	CTL Group (*n* = 76)	RT Group (*n* = 81)
**Time in hemodialysis (months)**		
Mean and SD.	55.47 ± 8.171	54.09 ± 11.05
Minimum	35.00	35.00
25% Percentile	49.25	44.00
Median	56.00	53.00
75% Percentile	61.00	64.00
Maximum	70.00	71.00
**Age (years)**		
Mean and SD.	66.33 ± 3.88	67.27 ± 3.24
Minimum	60.00	60.00
25% Percentile	63.25	66.00
Median	66.00	68.00
75% Percentile	70.00	69.00
Maximum	72.00	72.00
**BMI (kg/m^2^)**		
Mean and SD.	26.82 ± 2.90	27.30 ± 3.77
Minimum	21.65	20.93
25% Percentile	24.87	25.10
Median	27.23	27.40
75% Percentile	28.52	28.65
Maximum	33.61	34.60

Mann–Whitney. CTL, control group; RT, resistance training; BMI, body mass index.

**Table 3:** BDNF levels redox balance profile and handgrip strength at baseline and post-training between CTL and RT group.

Variables	Control Group (*n* = 76)		Resistance-Trained Group (*n* = 81)		*p*-Value
	Baseline	Post	Baseline	Post	
**BDNF (ng/mL)**					
Mean and SD.	14.40 ± 4.99	10.84 ± 5.94 ^a^	11.66 ± 5.20	19.60 ± 7.23 ^a,b^	0.0001
Minimum	4.380	−0.2600	1.360	4.650	
25% Percentile	12.10	6.378	6.390	15.31	
Median	15.34	10.10	12.35	20.23	
75% Percentile	16.98	15.46	16.37	24.35	
Maximum	31.45	24.45	19.35	34.30	
**TROLOX (uM)**					
Mean and SD.	627.0 ± 191.6	589.5 ± 195.9	596.2 ± 218.3	680.8 ± 225.2 ^b^	0.001
Minimum	200.0	204.0	211.0	232.0	
25% Percentile	497.0	451.0	415.5	490.5	
Median	643.0	622.5	647.0	734.0	
75% Percentile	761.8	755.3	792.5	878.5	
Maximum	894.0	899.0	882.0	1027	
**GSH (µM)**					
Mean and SD.	4.66 ± 1.93	5.00 ± 2.96	4.23 ± 1.84	9.33 ± 2.09 ^a,b^	0.0001
Minimum	1.130	0.0800	1.090	4.580	
25% Percentile	3.178	2.578	2.715	8.050	
Median	4.675	4.745	4.240	9.210	
75% Percentile	5.843	7.110	5.445	10.68	
Maximum	9.500	13.40	8.690	14.53	
**TBARS (nmol/mL)**					
Mean and SD	13.26 ± 2.45	13.66 ± 2.62	14.17 ± 2.39	11.06 ± 2.95 ^a,b^	0.0001
Minimum	9.410	8.790	9.220	3.850	
25% Percentile	11.19	11.70	12.05	8.735	
Median	12.65	13.43	14.63	11.38	
75% Percentile	15.69	15.40	16.36	13.03	
Maximum	18.64	20.53	18.73	16.58	
**Handgrip (kgf)**					
Mean and SD.	20.09 ± 5.19	19.75 ± 5.54	21.17 ± 4.38	27.17 ± 4.34 ^a,b^	0.0001
Minimum	12.00	10.00	12.00	12.00	
25% Percentile	16.00	16.25	18.00	25.00	
Median	19.00	19.50	22.00	27.00	
75% Percentile	23.00	23.75	25.00	30.50	
Maximum	30.00	32.00	30.00	37.00	

Kruskal–Wallis with Dunn’s multiple comparisons test. ^a^ *p* = 0.0001 vs. pre. ^b^ *p* = 0.0001 vs. CTL post-training. BDNF (ng/mL), brain-derived neurotrophic factor. TROLOX (µM), total antioxidant capacity; GSH (µM), glutathione; TBARS (nmol/mL), thiobarbituric acid reactive substances.

**Table 4:** Baseline and post-intervention Medical Outcomes Study—SF36 between control and resistance-trained group.

Medical Outcomes SF36	Control Group (*n* = 76)		Resistance-Trained Group (*n* = 81)		*p*-Value
	Baseline	Post	Baseline	Post	
**Physical functioning**					
Mean and SD.	70.17 ± 6.616	68.49 ± 7.057	69.19 ± 5.878	82.27 ± 6.166 ^a,b^	0.0001
Minimum	60.00	56.00	60.00	70.00	
25% Percentile	64.25	64.00	64.50	77.50	
Median	70.00	68.50	69.00	82.00	
75% Percentile	76.00	75.00	74.00	86.00	
Maximum	80.00	80.00	80.00	95.00	
**Physical role**					
Mean and SD.	75.43 ± 6.076	73.78 ± 6.378	74.63 ± 6.159	88.67 ± 6.253 ^a,b^	0.0001
Minimum	65.00	62.00	65.00	77.00	
25% Percentile	70.00	68.25	70.00	84.00	
Median	75.00	74.00	73.00	88.00	
75% Percentile	80.75	79.75	81.00	94.00	
Maximum	85.00	85.00	85.00	99.00	
**Pain**					
Mean and SD.	54.84 ± 7.005	53.50 ± 6.977	53.25 ± 7.680	71.32 ± 7.878 ^a,b^	0.0001
Minimum	42.00	38.00	42.00	58.00	
25% Percentile	48.50	48.00	46.00	65.00	
Median	56.00	54.00	53.00	71.00	
75% Percentile	62.00	60.00	60.00	78.00	
Maximum	65.00	65.00	65.00	86.00	
**General health**					
Mean and SD.	52.26 ± 6.363	50.89 ± 6.275	51.93 ± 6.249	63.90 ± 6.818 ^a,b^	0.0001
Minimum	42.00	38.00	42.00	51.00	
25% Percentile	47.00	46.00	47.00	58.00	
Median	52.50	51.00	51.00	64.00	
75% Percentile	58.00	57.00	57.00	69.50	
Maximum	62.00	63.00	62.00	77.00	
**Vitality**					
Mean and SD.	69.78 ± 6.168	68.34 ± 6.313	69.47 ± 6.044	76.99 ± 6.288 ^a,b^	0.0001
Minimum	60.00	57.00	60.00	65.00	
25% Percentile	64.00	63.00	64.50	72.00	
Median	70.00	68.50	69.00	77.00	
75% Percentile	75.75	74.00	74.00	82.00	
Maximum	80.00	80.00	80.00	90.00	
**Social function**					
Mean and SD.	63.20 ± 5.458	61.97 ± 5.535	62.94 ± 6.053	72.35 ± 6.118 ^a,b^	0.0001
Minimum	52.00	49.00	52.00	61.00	
25% Percentile	58.00	58.00	58.00	67.00	
Median	63.50	62.00	64.00	73.00	
75% Percentile	67.00	66.00	68.00	77.50	
Maximum	72.00	73.00	72.00	83.00	
**Emotional role**					
Mean and SD.	71.01 ± 5.375	69.64 ± 5.701	71.68 ± 5.922	81.31 ± 6.895 ^a,b^	0.0001
Minimum	62.00	59.00	62.00	68.00	
25% Percentile	66.00	65.00	67.00	75.00	
Median	71.00	69.50	72.00	82.00	
75% Percentile	75.00	74.75	77.00	87.00	
Maximum	80.00	80.00	80.00	94.00	
**Emotional well-being**					
Mean and SD.	62.38 ± 5.944	61.01 ± 6.245	61.06 ± 6.410	79.00 ± 6.624 ^a,b^	0.0001
Minimum	52.00	48.00	52.00	68.00	
25% Percentile	57.00	56.25	55.00	73.00	
Median	63.00	61.00	60.00	78.00	
75% Percentile	67.00	66.00	67.50	86.00	
Maximum	72.00	73.00	72.00	92.00	

Kruskal–Wallis with Dunn’s multiple comparisons test. ^a^ *p* = 0.0001 vs. pre. ^b^ *p* = 0.0001 vs. CTL post.

**Table 5:** Baseline and post-intervention Medical Outcomes Study—SF36 between control and resistance-trained group.

	Control Group (*n* = 76)		Resistance-Trained Group (*n* = 81)	
	Baseline	Post	Baseline	Post
Beck Depression Inventory				
Mean and SD.	26.83 ± 3.81	27.28 ± 4.35	28.10 ± 6.11	26.33 ± 6.28
Minimum	20.00	19.00	19.00	15.00
25% Percentile	24.25	24.00	24.00	22.00
Median	26.00	28.00	26.00	25.00
75% Percentile	29.00	29.00	31.50	30.00
Maximum	39.00	40.00	43.00	43.00
∆ Beck Depression score	0.45 ± 1.67		−1.76 ± 2.00 *	

Kruskal–Wallis with Dunn’s multiple comparisons test. * *p* = 0.0001 vs. pre ∆, variation between post –training and baseline.

## Data Availability

The data that support the findings of this study are available from the corresponding author upon reasonable request.
